# Medical News

**Published:** 1854-08

**Authors:** 


### MEDICAL NEWS.

Late arrivals from Barbadoes have brought us accounts of an outbreak of cholera in the island, unprecedented, perhaps, in the annals of that fearful pestilence. We have been favored by a gentleman, residing in the island, with the daily number of deaths for one month, in the Parish of St. Michael alone. Before giving this statement, however, it may not be uninteresting to give the population of the Parish, so that we may be able to compare the mortality with that of other places.

As would be expected, the disease has been most fatal among the colored and black population—not more than 5 per cent. of the deaths being whites. It is not known how it originated, but it is pretty certain, from all that we can learn, that it was not imported.

The census of the island, which was taken in 1851, and is now before us, gives the population of the Parish of St. Michael as 37,466, of which 5,313 are whites, the remaining 32,153 being colored and black.

We now give the list sent us:

| | | | | | |
|---|---|---|---|---|---|
| May 14, | 8 | May 25, | 33 | June 5, | 84 |
| " 15, | 10 | " 26, | 35 | " 6, | 133 |
| " 16, | 12 | " 27, | 38 | " 7, | 199 |
| " 17, | 19 | " 28, | 16 | " 8, | 132 |
| " 18, | 22 | " 29, | 27 | " 9, | 224 |
| " 19, | 24 | " 30, | 29 | " 10, | 226 |
| " 20, | 26 | " 31, | 36 | " 11, | 304 |
| " 21, | 27 | June 1, | 40 | " 12, | 296 |
| " 22, | 29 | " 2, | 43 | " 13, | 244 |
| " 23, | 30 | " 3, | 77 | " 14, | 270 |
| " 24, | 31 | " 4, | 93 | | |
| | | | Total, | | 2807 |

This gives a mortality of about 7½ per cent. per month.

That we may be able to compare this mortality with that of other places, we give a table below, taken, with a little alteration, from Dr. Baly's report on cholera, lately published. It may be stated that the usual annual mortality of the whole island is about 1 in 65.

*Table showing the Monthly Mortality from Cholera in several Epidemics in several Cities and Towns.*

| | London | | Paris | | Liverpool | Berlin | | | | Cologne | Stockholm |
|---|---|---|---|---|---|---|---|---|---|---|---|
| | 1832 | 1849 | 1832 | 1849 | 1849 | 1831 | 1832 | 1837 | 1848 | 1849 | 1834 |
| Months. | Deaths from Cholera. | Deaths from Cholera. | Deaths from Cholera. | Deaths from Cholera. | Deaths from Cholera. | Deaths from Cholera. | Deaths from Cholera. | Deaths from Cholera. | Deaths from Cholera. | Deaths from Cholera. | Deaths from Cholera. |
| January | | 292 | | | 5 | | 7 | | | | |
| February | 57 | 180 | | | 7 | | 1 | | | | |
| March | 880 | 40 | 90 | 573 | 18 | | | | | | |
| April | 335 | 9 | 12733 | 1929 | 19 | | | | | | |
| May | 50 | 24 | 811 | 4509 | 96 | | | | | | |
| June | 194 | 279 | 868 | 8669 | 424 | | | | | | |
| July | 1363 | 2555 | 2573 | 865 | 1085 | | | | 2 | 92 | |
| August | 1198 | 5368 | 969 | 1382 | 1575 | 2 | 5 | 942 | 261 | 259 | 169 |
| September | 670 | 5031 | 357 | 1142 | 874 | 577 | 29 | 1167 | 878 | 610 | 3416 |
| October | 336 | 337 | 62 | 115 | 62 | 562 | 220 | 141 | 360 | 203 | 52 |
| November | 27 | 20 | | | 2 | 133 | 114 | 16 | 39 | 10 | 7 |
| December | | 2 | | | 6 | 14 | 30 | | | | 21 |

The London Medical Times and Gazette of July 8th, 1854, contains a letter by Mr. James Turle, upon the "Treatment of Transverse Fractures of the Patella." The plan recommended consists in "so applying successive strips of adhesive plaster, that each shall gradually bring the displaced fragment nearer to its proper position," &c. By permission of Mr. Erichson, it had been successfully employed at University College Hospital.

We think it but proper to mention that this mode of treating fracture of the patella was first employed at the Pennsylvania Hospital by Dr. John Neill. A report of two cases so treated by him may be seen in the January number of this Journal.

---

*Abstract of Meteorological Observations for June,* 1854, *made at Philadelphia, Pa. Latitude* 39° 57′ 28″ *N., Longitude* 75° 10′ 40″ *W. from Greenwich.* By PROF. JAMES A. KIRKPATRICK.

| 1854. June. | Barometer. Daily Mean | Barometer. Mean Daily Range. | Thermom. Daily Mean | Thermom. Mean Daily Range | Dew Point 2 P M. | Rain. | Prevailing Winds. | General Remarks. |
|---|---|---|---|---|---|---|---|---|
| | Inches. | Inches. | Deg. | Deg. | Deg. | Inch. | Points. | |
| 1 | 30.062 | .102 | 58.5 | 3.8 | 37.7 | | (Var.) | Cloudy. *Therm. lowest* 49°. |
| 2 | 30.065 | .058 | 65.0 | 5.8 | 42.3 | | (Var.) | Clear. *Barom. highest* 30.098 in. |
| 3 | 29.971 | .094 | 68.5 | 4.8 | 42.5 | | W. | Clear. |
| 4 | 29.919 | .052 | 69.7 | 0.8 | 53.7 | | SW. | Clear. |
| 5 | 29.841 | .078 | 73.5 | 1.5 | 55.3 | | SW. | M. cloudy; aft. and evening clear. |
| 6 | 29.792 | .049 | 75.5 | 3.2 | 56.0 | | SSW. | Cloudy. |
| 7 | 29.687 | .102 | 72.0 | 3.8 | 69.0 | 0.416 | SE. | Cloudy. Aft. and ev. Rain. |
| 8 | 29.582 | .108 | 74.5 | 3.0 | 63.3 | | (Var.) | Cloudy. *Barom. lowest* 29.549. |
| 9 | 29.754 | .172 | 66.5 | 7.0 | 47.7 | | SW. | Cloudy. |
| 10 | 29.889 | .135 | 64.3 | 2.5 | 51.7 | | W. | M. and ev. clear; aft. cloudy. |
| 11 | 29.915 | .026 | 69.3 | 3.0 | 54.3 | 0.035 | SW. | Cloudy. |
| 12 | 29.842 | .073 | 70.0 | 1.5 | 57.3 | | S. | Cloudy. |
| 13 | 29.712 | .130 | 63.5 | 5.3 | 60.3 | 0.866 | (Var.) | Cloudy. Aft. thunder storm. |
| 14 | 29.679 | .033 | 71.5 | 7.7 | 53.3 | | W. | Clear. |
| 15 | 29.698 | .019 | 73.5 | 2.7 | 53.7 | | (Var.) | M. and aft. cloudy; ev. clear. |
| 16 | 29.801 | .103 | 76.5 | 1.5 | 49.0 | | NW. | M. and aft. cloudy; ev. clear. |
| 17 | 29.993 | .192 | 70.0 | 3.8 | 52.0 | | S. | M. and aft. cloudy; ev. clear. |
| 18 | 29.923 | .070 | 73.0 | 4.7 | 62.0 | | SSW. | M. and ev. clear; aft. cloudy. |
| 19 | 29.834 | .089 | 78.5 | 5.0 | 58.7 | | SW. | M. and aft. cloudy; ev. clear. |
| 20 | 29.811 | .023 | 79.0 | 1.8 | 42.7 | | (Var.) | M. and aft. cloudy; ev. clear. |
| 21 | 29.782 | .029 | 79.3 | 1.3 | 49.8 | 0.758 | (Var.) | M. clear; aft. and ev. cloudy. |
| 22 | 29.828 | .062 | 64.0 | 16.2 | 61.3 | | ENE. | Cloudy. |
| 23 | 29.833 | .059 | 66.5 | 5.8 | 60.3 | 0.012 | SW. | Cloudy. |
| 24 | 29.730 | .103 | 73.0 | 6.8 | 55.3 | | NNW. | Cloudy. |
| 25 | 29.801 | .078 | 70.0 | 4.3 | 45.7 | | NW. | Cloudy. |
| 26 | 29.892 | .112 | 69.0 | 3.3 | 54.7 | | SW. | Cloudy. |
| 27 | 29.811 | .081 | 81.5 | 11.7 | 61.3 | | (Var.) | Clear. *Therm highest* 96°. |
| 28 | 29.750 | .072 | 80.5 | 2.3 | 69.7 | 0.081 | (Var.) | Cloudy; aft. thunder storm. |
| 29 | 29.784 | .072 | 77.7 | 4.3 | 51.7 | | (Var.) | M. and aft. clear; ev. cloudy. |
| 30 | 29.728 | .055 | 74.5 | 3.0 | 72.7 | 1.273 | (Var.) | M. and aft. cloudy; ev. clear. |
| Means for 1854 | 29.824 | .081 | 71.6 | 4.4 | 54.8 | 3.441 | S. 76° W. 52—100. | |
| Means for 1853 | 29.987 | | 73.6 | | 54.2 | 1.050 | | |
| Means for 1852 | 29.848 | | 71.6 | | 50.9 | 4.500 | | |
| Means for 3years | 29.886 | | 72.3 | | 53.3 | 2.997 | S. 71° W. 47—100. | |

The extreme range of the barometer during the month was 0.549 of an inch, and of the thermometer 47°.

